# Intelligent Segmentation Algorithm for Diagnosis of Meniere's Disease in the Inner Auditory Canal Using MRI Images with Three-Dimensional Level Set

**DOI:** 10.1155/2021/2329313

**Published:** 2021-07-20

**Authors:** Ting Liu, Ying Xu, Yujuan An, Hongzhou Ge

**Affiliations:** ^1^Department of Otolaryngology, Qingdao Hospital of Traditional Chinese Medicine (Qingdao Hiser Hospital), Qingdao 266034, Shandong, China; ^2^Department of Intravenous Infusion Center, Qingdao Hospital of Traditional Chinese Medicine (Qingdao Hiser Hospital), Qingdao 266034, Shandong, China

## Abstract

This paper aimed to explore segmentation effects of the magnetic resonance imaging (MRI) images of the inner auditory canal of patients with Meniere's disease under the intelligent segmentation method of the inner ear based on three-dimensional (3D) level set (IS3DLS). The statistical shape model and the level set segmentation algorithm were combined to propose the IS3DLS. First, the shape training samples of the inner ear model were determined, and the results were manually segmented to further obtain region of interest (ROI) of the inner ear. The IS3DLS was employed to accurately segment MRI images of the inner auditory canal of patients with Meniere's disease. The segmentation performance of IS3DLS was compared with the expert manual segmentation method and the region growth level set-based segmentation algorithm. Results showed that Matthews correlation coefficient (MCC), Dice similarity coefficient (DSC), false positive rate (FPR), and false negative rate (FNR) of this algorithm were 0.9599, 0.9594, 0.0325, and 0.03655, respectively. Therefore, the IS3DLS could achieve good segmentation effect in MRI images of the inner auditory canal of patients with Meniere's disease, which was helpful for diagnosis and subsequent treatment of Meniere's disease.

## 1. Introduction

Meniere's disease is an idiopathic inner ear disease, and the inner ear mainly includes bony and membranous labyrinths, which locates between the thigh chamber and the bottom of the inner ear canal. The main pathological change of this disease is membrane labyrinth hydrops, which is clinically manifested as recurrent rotating vertigo, fluctuating hearing loss, tinnitus, and ear fullness. It mostly occurs in young and middle-aged people aged 30–50 years, and the incidence is about 0.2–0.5% [1]. Meniere's disease is mostly caused by various infections, injuries, otosclerosis, tumors, leukemia, autoimmune diseases, and genetic factors. Clinical examination often applies the imaging detection, and MRI of the inner ear membrane labyrinth under special contrast can show the narrowing of the endolymphatic vessels in some patients [2]. MRI is a type of tomography that adopts the magnetic resonance phenomena to obtain electromagnetic signals from the human body, so as to reconstruct the information of human body. It can display the distribution of a certain physical quantity in panic and weight loss and can obtain tomographic images and three-dimensional images in any direction. Besides, it is featured with the absence of ionizing radiation, clear soft tissue structure, and multisequence imaging. The shortcoming is that the spatial resolution is not high [3].

The regional growth level set segmentation algorithm is to initially determine the inner ear contour through the traditional regional growth cutting, to remove the inner ear boundary noise, and to repair the inner ear boundary by the adaptive curvature threshold method. Finally, the distance regularized level set evolution (DRLSE) model in the level set method is applied to accurately segment the inner ear region [4]. This method can effectively prevent missed detection of image edges and can process images of various types of lesions. However, the accuracy and operability of this method need to be improved [5]. Statistical shape models are the models that employ the high-resolution images as training samples and are compared with the images to be tested, so as to obtain accurate solutions through appropriate registration methods [6]. The level set segmentation algorithm adopts the evolution of a 3D surface to represent the evolution of a 2D curve. It means that the continuous evolution of the curve motion is applied to find the boundary of the image until the target contour is found, and then, the curve stops moving. The curve moves along each section of the image, slices are taken from different sections, and then, a closed curve is obtained. As time goes by, the level set is changed and the vision domain is calculated. The level set method can have a good image segmentation effect in order to obtain a corresponding shape for the contour extraction [7, 8].

To sum up, the statistical shape model was compared with the level set segmentation algorithm in order to improve the efficiency of doctors' diagnosis of inner auditory canal MRI images of Meniere's disease patients, so IS3DLS was put forward in this study. There was a comparison on segmentation results of different segmentation algorithms (this algorithm, the region growth level set segmentation algorithm, and the expert manual segmentation method). Therefore, the optimal segmentation method was selected and evaluated through the above.

## 2. Experimental Methods

### 2.1. Establishment of Research Data

68 patients with Meniere's disease were selected as the research objects of this study, who were diagnosed and treated at hospital from October 2017 to April 2020 with symptoms of tinnitus. They were 18–62 years old, including 38 males and 30 females. The criteria for inclusion were defined to include patients who were in line with the diagnosis basis and efficacy evaluation of Meniere's disease of the Otolaryngology Branch of the Chinese Medical Association. (1) There were 2 or more episodes of vertigo, each lasting from 20 minutes to 12 hours. (2) At least one hearing test confirmed low-medium and low-frequency hearing loss and fluctuating hearing loss. The criteria for exclusion were defined to include patients who suffered from Meniere's disease and who had received endolymphatic sac surgery, cochlear implant surgery, and femoral gentamicin injection. Vertigo caused by other diseases, such as benign paroxysmal positional vertigo, labyrinthitis, and vestibular neuritis, was excluded. This experiment had been authorized by the Ethics Committee of hospital, and all the research objects and their family members had signed the consent forms.

### 2.2. Establishment of the Statistical Shape Model of the Inner Ear

First, all training samples were placed in the same coordinate space. Platts analysis was used for alignment of the corresponding training sample sets with the same number of feature points. Under a 2D sample space, the sum of squared errors between each shape sample *S*_*i*_ and the average shape *S* was expressed as *D* : *D* = |(*S*_*i*_–*S*)^2^|. The initial average shape was a sample image randomly specified from the training sample set. *D* expressed the Platts distance, which was denoted by *P*_*d*_. Thus, the Platts distance between any two shapes *S*_*i*_ and *Sj* could be calculated as follows:

(1) $$\begin{array}{c} P_{d}=\sum_{m=1}^{n}\left|{\left(s_{i}-s_{j}\right)}^{2}\right|=\sum_{m=1}^{n}\left|{\left(x_{im}-x_{jm}\right)}^{2}+{\left(y_{im}-y_{jm}\right)}^{2}\right|. \end{array}$$

In (1), *n* stood for the number of feature points contained in each shape.

Under the expansion condition of high-dimensional space, it was necessary to align any two shape samples. First, the center of each shape should be calculated, and all shapes should be unified in size and expressed by the Euclidean distance equation, as shown in

(2) $$\begin{array}{c} E\left(S\right)=\sqrt{\sum_{m=1}^{n}\left[{\left(x_{m}-\bar{x}\right)}^{2}+{\left(y_{m}-\bar{y}\right)}^{2}\right]}. \end{array}$$

The centers of the two shapes should be aligned, and the singular value decomposition method was applied to align the rotation direction. Then, the final aligned sample was obtained by minimizing the Platts distance.

### 2.3. Measurement of the Image by Three-Dimensional Registration Technology

**Figure 1:**
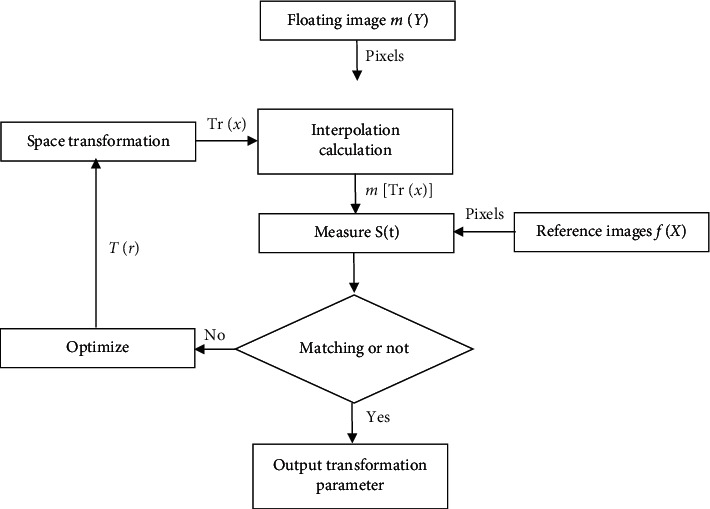
Flow chart of image registration.

According to the image registration process in Figure 1, the reference and floating images were first input, and the reference image was transformed into geometric coordinates according to the given initial transformation parameters. Then, the floating image was calculated through interpolation to obtain the value of the new coordinate area. The similarity of reference image and the floating images after the difference should also be calculated. If the similarity met the preset registration requirements, the change parameters were output to obtain the interpolated image of the best floating image. However, the optimization would be continued if the registration requirements were not met. The transformation parameters were changed until the final parameters that met the requirements were obtained.

### 2.4. Acquisition of the Region of Interest in the Inner Ear

In this study, registration technology was employed to automatically locate and acquire the ROI of the inner ear. The 3D rigid body transformation was selected as the transformation model. Furthermore, the rigid body transformation included translation and rotation.

(3) $$\begin{array}{c} \left(X_{m},Y_{m},Z_{m}\right)=A\left(X_{f},Y_{f},Z_{f}\right)+b=\text{AF}+b. \end{array}$$

In (3), (*X*_*m*_, *Y*_*m*_, *Z*_*m*_) represented the spatial position coordinates of the registered image and (*X*_*f*_, *Y*_*f*_, *Z*_*f*_) stood for the spatial position coordinates of the target image to be transformed. *A* and *b* expressed the rotation matrix and the translation vector in turn, and the constraints of the matrix were *A*^*T*^ = I and det*A*=1. Besides, AT was the transpose of *A*, and *I* meant the identity matrix. The mutual information was chosen in this study as the similarity metric. For a given image *X* and *Y*, their mutual information was expressed as follows:

(4) $$\begin{array}{c} \text{MI}\left(X,Y\right)=H\left(X\right)+H\left(Y\right)-H\left(X,Y\right). \end{array}$$

In (4), *H(X)*, *H(Y)*, and *H(X*, *Y)* were the entropy *X*, entropy *Y*, and their joint entropy, respectively.

(5) $$\begin{array}{c} H\left(X,Y\right)\leq H\left(X\right)+H\left(Y\right). \end{array}$$

When *X* and *Y* were independent of each other, the joint entropy was equal to the sum of their respective entropy. When *X* and *Y* were not independent of each other, the joint entropy was less than the sum of their respective entropy. The above was expressed in (5).

The gradient descent method was selected as the optimization algorithm of the registration process, and the objective function was optimized by an iterative method to achieve the best conditions. Then, the cubic polynomial interpolation method was adopted to make the nonlinear difference of the voxel points, so that the final registered image could be obtained. Thus, the ROI of inner ear could be automatically located and identified. The algorithm of the difference result could be calculated as

(6) $$\begin{array}{c} v\left(x,y\right)=\sum_{i=0}^{3}\sum_{j=0}^{3}a_{ij}x^{i}y^{i}. \end{array}$$

### 2.5. Segmentation of the Three-Dimensional Level Set Algorithm

**Figure 2:**
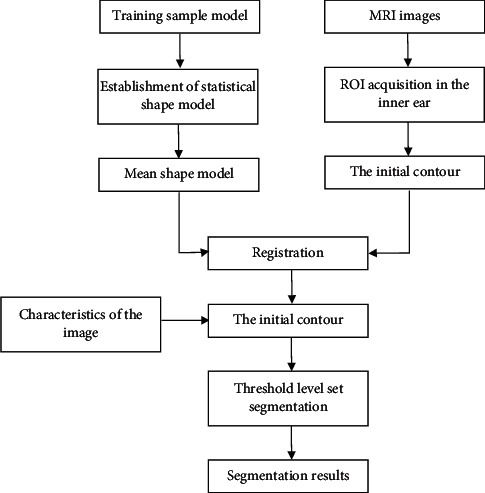
Flow chart of inner ear level set segmentation based on the statistical shape model.

The MRI image of the inner ear of patient with Meniere's disease was segmented through the method proposed in this study.  Figure 2 indicates the segmentation process of IS3DLS. The main steps were as follows. First, the training set samples were collected to create the inner ear statistical shape model, and then, the average shape model was calculated. Second, the inner ear MRI image was for weighting and the ROI of inner ear was extracted. Third, the inner ear ROI and the average shape model were registered to obtain the initial contour. Fourth, the level set was applied to segment the initial contour and the characteristic image to get the result after the segmentation.

### 2.6. Evaluation Indicators of Inner Ear Segmentation Accuracy

Taking the expert manual segmentation as the standard, the true position (TP) and the false position (FP) were employed to measure the error between the regions obtained by the two segmentation algorithms. Moreover, the average minimum Euclidean distance (AMED) and Hausdorff distance (HD) were adopted to determine the difference between the algorithm segmentation contour and the manual segmentation contour.

The four indicators (MCC, DSC, FPR, and FNR) were adopted in this study to evaluate the accuracy of the segmentation results. The manual segmentation result has been forwarded as a standard. The IS3DLS, regional level set segmentation, and expert manual segmentation were compared, and the comparison results were calculated. In addition, DSC and MCC verified the similarity between the results of different algorithm segmentation and expert manual segmentation by calculating the overlap ratio of pixels. The calculation result was near 1, which meant that the calculation result of IS3DLS was closer to the result of expert manual segmentation algorithm, and the accuracy was higher. FPR and FNR represented the oversegmentation and undersegmentation in sequence. The closer the value was to 0, the lower the degree of error in segmentation was and the better the accuracy was.

### 2.7. Statistical Analysis

All measurement data were expressed as mean ± standard deviation (SD), and SPSS 21.0 software package was used for statistical processing of data. Analysis of variance (ANOVA), *t*-test, and chi-square test were used for intragroup comparison, intergroup comparison, and the count data comparison, respectively. In addition, *P* < 0.05 indicated the difference was statistically substantial.

## 3. Experimental Results

### 3.1. MRI Images of the Inner Auditory Canal of Patients with Meniere's Disease

**Figure 3:**
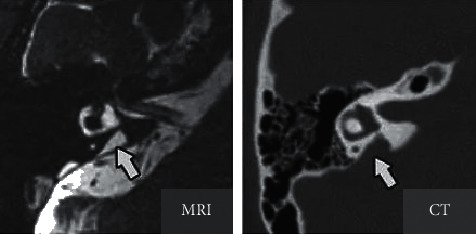
The images of the inner ear of one child patient with Meniere's disease.

Figure 3 shows the MRI and computed tomography (CT) images of the inner ear of one patient. This patient was diagnosed with enlarged vestibular aqueducts; the area indicated by the arrow presented the abnormal enlargement of endolymphatic vessels and sac, thus confirming that the patient suffered from Meniere's disease.

### 3.2. Experimental Results of Level Set Segmentation of the Inner Ear Based on the Statistical Shape Model

**Figure 4:**
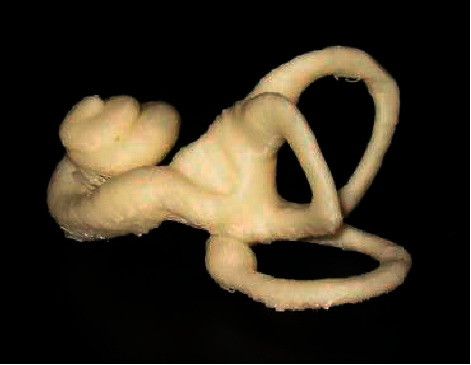
Modeling results of the statistical shape model of the inner ear.

As shown in Figure 4, the inner ear shape samples were used for statistical analysis, so that the inner ear shape model was established. Then, the average shape of the inner ear was obtained through the statistical model.

**Figure 5:**
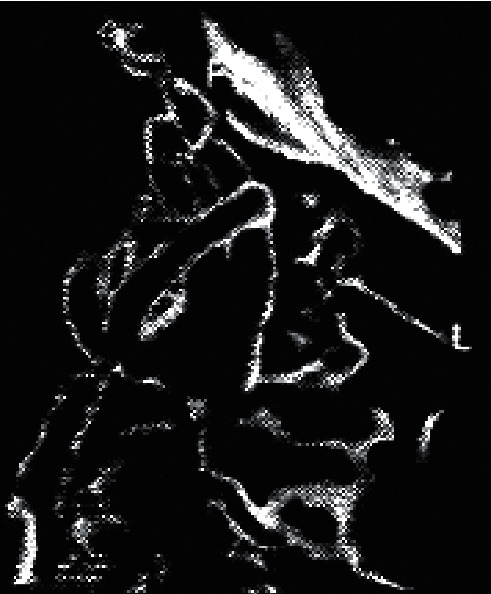
Drawing result of the inner ear ROI.

**Figure 6:**
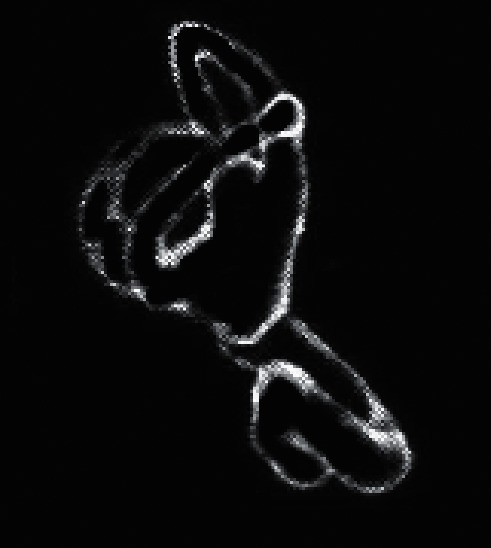
Result of the segmentation algorithm in this study.

Based on the inner ear average shape model as the initial contour, the level set segmentation algorithm was used for segmentation. One of the images was arbitrarily selected as a reference, the other images were extracted through registration technology to take the inner ear ROI out, and the inner ear ROI was segmented through level set evolution to obtain the image (Figure 5). The inner ear was drawn through the segmentation algorithm proposed in this study to obtain Figure 6. Through comparison, it was found that the algorithm proposed in this study showed clear display of the vestibule, cochlea, and semicircular canal, and the segmentation was accurate. Thus, the results were basically consistent with the actual structure of the inner ear.

The comparison results of the three segmentation methods are shown in Table 1. The measurement indicators in Table 1 were evaluated from the accuracy and error of the segmentation results. The results revealed that the segmentation algorithm in this study had a good segmentation effect, and the accuracy and error rates were close to 1 and 0 in turn. It indicated that the results of segmentation algorithm proposed in this study were similar to the manual result of experts.

### 3.3. Comparison on the Results of Different Segmentation Algorithms

Table 2 discloses that the other two segmentation algorithms had a high TP value (>90%) compared with expert manual segmentation, and the IS3DLS was higher than the region growth set segmentation. The FP value of IS3DLS was obviously smaller than that of the region growth set segmentation (*P* < 0.05). The AMED and HD values of IS3DLS decreased sharply in contrast to the region growth set segmentation. The difference in HD values *pf* patients from the two groups was statistically marked (*P* < 0.05).

Table 3 indicates the comparison results of the 3D surface gray matter of the three segmentation algorithms. By comparing with expert manual segmentation, the accuracy indicators of IS3DLS and the segmentation under the region growth level set were compared, and the results are shown in Table 3. MCC stood for the similarity of the two data. From the table, IS3DLS had a higher overlap rate and closer results with expert manual segmentation in contrast to the region growth set segmentation. DSC represented the segmentation effect. When the DSC value was greater than 0.8, the algorithm segmentation effect is considered good. From the table below, the segmentation effect of the two segmentation algorithms was good, but the segmentation effect of IS3DLS was better. PPR meant the false positive rate, which was employed to indicate undersegmentation, FNR expressed the false negative rate, which indicated oversegmentation, and FPR and FNR both showed the error of the segmentation effect. The FPR and FNR values of IS3DLS were lower steeply than the values of region growth set segmentation (*P* < 0.05). It further demonstrated the accuracy of the segmentation method in this study.

### 3.4. Analysis on Complexity of Different Segmentation Algorithms

Table 4 shows the running time of the region growth set segmentation and the segmentation algorithm proposed in this study. The running time of the region growing set segmentation was 37.15 s, and the running time of IS3DLS was 23.53 s. Thus, the running time of IS3DLS was markedly less than that of the region growth set segmentation (*P* < 0.05).

## 4. Discussion

The cause of Meniere's disease is still unclear, which may be related to the balance of endolymph production and absorption. The structure of the inner ear has complex changes [9], and 3D-weighted water imaging sequences are often used for multiplanar reconstruction. The above is adopted for MRI examinations of inner ear diseases, thin-slice scanning of auditory nerves, and water imaging of inner ear labyrinth [10]. The advantage of MRI is that it does not produce bony false shadows and is suitable for diseases of various systems throughout the body. However, the imaging time of MRI is long and the spatial resolution is low, which is only 2 mm according to statistics [11]. Medical image segmentation plays a vital role in digital medical research and the formulation and implementation of treatment plans. Noise and contrast have critical effects on image segmentation. Traditional segmentation algorithms are difficult to obtain satisfactory results and run for a long time. Although the expert manual segmentation method has a relatively high accuracy rate, it is time-consuming and challenging, and it is difficult for inexperienced doctors to accurately judge [12, 13].

There are more and more applications based on the level set method in image segmentation, which can incorporate images of different shapes into the energy function, so as to finally obtain the shape of the contour represented by the zero level function set. A level set segmentation algorithm is proposed based on differential geometry. When segmenting the images of ultrasound, CT, and MRI, this method can effectively segment the blurred area or partially missing lesions of the image boundary. The level set method has high segmentation accuracy [14, 15]. Statistical shape model is a powerful visualization and quantification tool that can connect geometric pattern with functional pattern. By testing the statistical shape model, the features of the patient image are obtained [16].

At present, the idea that the histopathologic change of Meniere's disease is endolymphatic hydrops is clinically recognized, but the cause of the hydrops is not fully understood pathologically in Meniere. Fiorino et al. [17] believed that endolymphatic hydrops was related to disease grade and VEMP, and the more severe the endolymphatic hydrops was, the worse the hearing was. However, the correlation between endolymphatic hydrops presented by MRI in Meniere's disease and other examination indicators was not confirmed yet. The experimental results of this study showed that MRI could detect abnormal enlargement of endolymphatic vessels and sacs, thereby diagnosing patient as Meniere's disease. Through statistical analysis of all training samples, a statistical shape model of the inner ear could be obtained. The inner ear ROI obtained by 3D registration technology was similar to the drawing results of the inner ear ROI. Compared with expert manual segmentation, the MCC and DSC values of IS3DLS were 0.9599 and 0.9594 in turn, and the values of the region growth set segmentation were 0.8693 and 0.8721, respectively. Although both segmentation methods had good segmentation results, the method proposed in this study was closer to 1, and its FPR and FNR were 0.0325 and 0.0365, respectively. Compared with the FPR and FNR of the region growth set segmentation (0.1402 and 0.0718), those of the method proposed in this study were lower, indicating that the IS3DLS was better. Moreover, the running time of IS3DLS was 23.53 s, which was extremely lower than the time of the regional growth set segmentation (37.15 s) (*P* < 0.05).

## 5. Conclusion

It was found that MRI images of the inner auditory canal could diagnose patient as Meniere's disease. The inner ear ROI obtained by the 3D registration technology was similar to the drawn inner ear ROI. The IS3DLS and the region growth set segmentation had good segmentation results. Compared with the region growth set segmentation, the segmentation effect of IS3DLS was closer to expert manual segmentation, and the error was smaller. The IS3DLS had faster speed and saved time, and its performance was related to the size of the training sample. When the training sample size is small, there is a certain error. However, it is difficult to obtain a large number of training samples in real experiments, so the error of the test is even greater when the shape of the image to be tested differs greatly from the selected training sample. It is hoped that more advanced segmentation algorithms will be introduced in the future to further improve the segmentation effect of MRI images and increase the application value of computer-assisted therapy in clinical medicine. The results of this study can provide an experimental basis for the investigation of the segmentation algorithm of MRI images of the inner auditory canal of Meniere's disease patients, thereby promoting the resolution of MRI images.

## Figures and Tables

**Table 1:** Performance test results of IS3DLS.

Group	MCC	DSC	FPR	FNR	*D*_mean_ (mm)	*D*_max_ (mm)
*A*	0.9532	0.9483	0.0353	0.0778	0.1573	1.1144
*B*	0.9758	0.9629	0.0517	0.0821	0.1424	0.9151
*C*	0.9442	0.9535	0.0125	0.0567	0.1842	0.7846
*D*	0.9431	0.9723	0.0215	0.0551	0.1165	0.8782
*E*	0.977	0.9682	0.0419	0.0614	0.1792	1.1286
*F*	0.9661	0.9512	0.0321	0.0977	0.1426	1.2379
Average	0.9599	0.9594	0.0325	0.0718	0.1537	1.0098

**Table 2:** Comparison of results of segmentation algorithms influenced by MRI.

Algorithm	TP	FP	AMED	HD
IS3DLS	97.81	1.52^*∗*^	0.78	2.15^*∗*^
Region growth set segmentation	96.36	2.78	0.83	2.98
*P*	>0.05	<0.05	>0.05	<0.05

Note: the symbol ^*∗*^ means that the difference was statistically substantial in contrast to the region growth set segmentation algorithm (*P* < 0.05).

**Table 3:** Quantitative analysis results of evaluation indicators in different algorithms.

Algorithm	MCC	DSC	FPR	FNR
IS3DLS	0.9599^*∗*^	0.9594^*∗*^	0.0325^*∗*^	0.0365^*∗*^
Region growth set segmentation	0.8693	0.8721	0.1402	0.0718
*P*	<0.05	<0.05	<0.05	<0.05

Note: the symbol ^*∗*^ shows there was a statistically obvious difference in contrast to the region growing set segmentation algorithm (*P* < 0.05).

**Table 4:** Comparison on running time of different algorithms.

Algorithm	Running time (s)
IS3DLS	23.53^*∗*^
Region growth set segmentation	37.15
*P*	<0.05

Note: the symbol ^*∗*^ reveals that the difference was statistically marked in contrast to the region growth set segmentation algorithm (*P* < 0.05).

## Data Availability

No data were used to support this study.
